# Computational Study of Graphene Quantum Dots (GQDs) Functionalized with Thiol and Amino Groups for the Selective Detection of Heavy Metals in Wastewater

**DOI:** 10.3390/molecules30244661

**Published:** 2025-12-05

**Authors:** Joaquín Alejandro Hernández-Fernández, Juan Sebastian Gómez Pérez, Edgar Marquez

**Affiliations:** 1 Chemistry Program, Department of Natural and Exact Sciences, San Pablo Campus, Universidad de Cartagena, Cartagena de Indias D.T. y C., Cartagena 130015, Colombia; 2 Department of Natural and Exact Science, Universidad de la Costa, Barranquilla 080002, Colombia; 3 Grupo de Investigación GIA, Fundacion Universitaria Tecnologico Comfenalco, Cr 44 D N 30A, 91, Cartagena 130015, Colombia; 4 Grupo de Investigaciones en Química Y Biología, Departamento de Química Y Biología, Facultad de Ciencias Básicas, Universidad del Norte, Barranquilla 081007, Colombia; ebrazon@uninorte.edu.co

**Keywords:** graphene quantum dots, adsorption, computational, heavy metals, selective detection, functionalization, contaminants, nanomaterials

## Abstract

Given the growing interest in contaminant detection, research has addressed the functionalization behavior of graphene quantum dots (GQDs) with thiol (-SH) and amino (-NH_2_) groups to optimize and improve the selective detection of heavy metals in wastewater. Implementing Density Functional Theory (DFT), the interactions between the functionalized GQDs and hydrated metals such as Cr, Cd, and Pb were simulated. The results showed that GQDs with thiol groups exhibited a high affinity for metals such as Pb and Cd, with an energy gap (Eg) of 0.02175 eV in the interaction with Pb, showing optimized reactivity. On the other hand, amino-modified GQDs presented a higher Eg, indicating a lower reactivity and efficacy in contaminant identification. Furthermore, this study evaluated electronic properties such as the energy gap and total dipole moment (TDM), resulting in the -SH-functionalized GQDs showing a higher TDM, which presented a greater interaction capacity with these metals. Likewise, the electrostatic potential maps (MEPs) provided information on the charge distribution when adsorbing metals, an important parameter to understand electronic interactions. These results showed that the modification of GQDs improved the detection of heavy metals, although limitations in the DFT method used are recognized and the need for experimental studies is suggested to validate the results and investigate other functional modifications.

## 1. Introduction

In recent decades, the development of alternatives for heavy metal detection has gained significant importance due to multiple environmental and human health problems caused by various industries, with mining and manufacturing being some of the main ones [1,2,3]. These industries release highly harmful metals such as mercury (Hg), arsenic (As), cadmium (Cd), and lead (Pb), among others, which are transported through the acid drainage of mines, contaminating soils and water bodies [4,5]. These metals are persistent in the environment and tend to bioaccumulate in ecosystems, affecting human health [5,6]. They also have a high affinity for biomolecules such as proteins, lipids, and nucleic acids [7,8,9], which can cause cell dysfunction and oxidative stress, as some heavy metals catalyze reactions that generate free radicals [6].

The growth of industries close to bodies of water has intensified heavy metal pollution, increasing health risks for the population [1,2]. However, these pollutants not only come from human activities but also from nature due to geographical phenomena such as volcanic eruptions, rock erosion, and leaching into rivers, lakes, and oceans [1,3,4]. Studies have shown that concentrations of heavy metals in lakes and rivers in North America and Europe are lower than in Africa, South America, and Asia [5]. In terms of average concentration per continent, Africa has the highest levels of iron (2012.82 μg/L) and aluminum (945.48 μg/L), followed by Asia with iron (3152.78 μg/L) and aluminum (3103.88 μg/L). In Europe, North America, and South America, the predominant metals also include iron and aluminum, although in different proportions [5].

**Figure 1:**
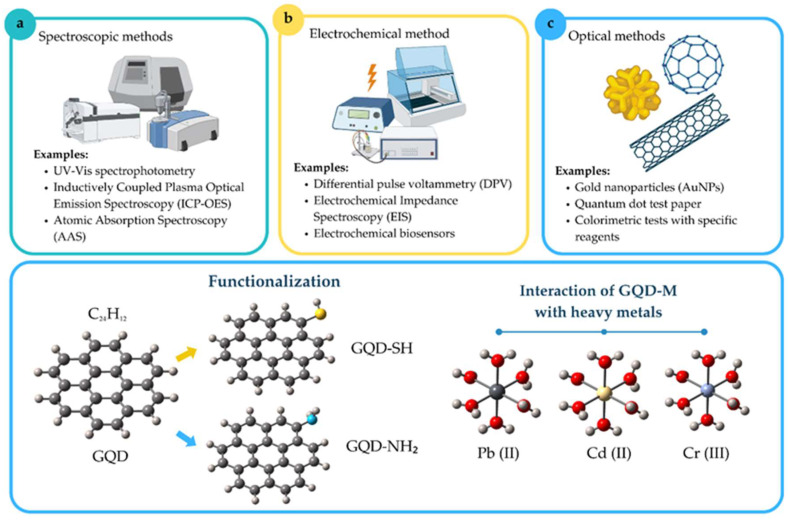
Heavy metal detection methods and functionalization of GQDs. (**a**) Spectroscopic methods include UV-Vis, ICP-OES, and AAS for heavy metal quantification. (**b**) Electrochemical methods: DPV, EIS, and biosensors for sensitive and selective detection. (**c**) Optical methods: Use of AuNPs, test paper with GQDs, and colorimetric tests for rapid detection.

Given this problem, various strategies have been developed for the detection of heavy metals in water bodies, which can be classified into three main categories: spectroscopic, electrochemical, and optical methods [2,6,7,8,9] (Figure 1). Traditional methods, such as atomic absorption spectroscopy (AAS) [10], anodic redissolution voltammetry (ASV) [11], and atomic plasma emission spectroscopy (ICP-AES) [12], are highly accurate and sensitive. However, they require expensive instrumentation and complex sample treatments [6,13]. In response to these limitations, modern methods based on electrochemical and electronic analysis have been developed in recent years, although the main limitations are low selectivity, instability, complex in situ sampling, and reduced compatibility in an aqueous environment [14]. In this context, optical methods have gained popularity due to their high compatibility in an aqueous environment, low cost, and being simple, fast, efficient, highly sensitive, and selective [15]. In this regard, the use of nanomaterials has been highlighted as a promising strategy for heavy metal detection [16,17,18,19]. Nanomaterials such as fullerenes, carbon nanotubes (CNTs), graphene oxide (GO), and quantum dots (QDs) have been extensively studied due to their tiny size (~10^−9^), high adsorption capacity, and improved electronic and optical properties [20,21]. In particular, graphene quantum dots (GQDs) have shown great potential for heavy metal detection due to their highly active electrical properties, facilitating efficient charge transfer and large surface areas [22]. GQDs also exhibit intrinsic fluorescence, making them critical tools for optical, biochemical, and environmental sensor applications [23,24]. Their ability to emit light under excitation, combined with their chemical versatility, makes them ideal for the selective detection of heavy metals and organic compounds [25,26].

Recently, the modification of GQDs has been explored, such as the doping of heteroatoms, creation of nanocomposites with inorganic or polymeric materials, and adjustment of the size and morphology of GQDs [27]. This involves improving the optical, chemical, and electronic properties, allowing their use in a wide variety of applications [28]. In a study, functionalized GQDs with BMIM^+^ (BMIM^+^-GQDs) were synthesized by the electrochemical cutting of three-dimensional graphene (3D) using 1-butyl-3-methylimidazoliohexafluorophosphate (BMIMPF6) as an electrolyte, achieving the efficient detection of Fe^3+^ [29]. Also, in 2012, a method to detect Cd^2+^ was developed using greenish-yellow luminescent GQDs (gGQD) obtained from nanosheets of graphene oxide (GO) by a microwave-assisted process under acidic conditions. Based on the intense electrochemiluminescence (ECL) of these nanomaterials, a novel ECL sensor for the detection of Cd^2+^ was proposed [30]. In 2015, research was carried out focusing on the modification of GQDs to improve their capacity to detect heavy metals such as Hg, Pb, and cadmium (Cd), obtaining as a result that the modification with thiol groups presented a high affinity for heavy metals, which is explained by a greater sensitivity and selectivity in detection [31,32].

GQDs with thiol (-SH) and amino (-NH_2_) groups have shown a significant increase in their performance in metal detection [27,33]. The incorporation of -SH groups in the structure of GQDs is effective for the capture and detection of metal ions due to the strong affinity of sulfur to metals such as Hg, Pb, and Cd [33,34,35,36]. On the other hand, the -NH_2_ group improves the solubility and stability of GQDs in aqueous solutions, as well as promoting their interaction with metal ions through coordination bonds and electrostatic attraction [36,37,38]. These functional groups facilitate the formation of covalent bonds with metals, which results in changes in the optical and electronic properties of GQDs and allows their use in high-precision fluorescent or colorimetric sensors [39,40].

To optimize cost and accuracy, computational research on materials such as GQDs facilitates the molecular analysis of requirements for incorporating functional groups [41,42,43]. By simulating the electronic structure, it is possible to anticipate its stability, interactions with metal ions, and alterations in its optical and electronic properties, thus reducing the need for expensive and extensive experimental tests [42,43,44]. Several computational studies have shown that the structural stability and electronic properties of GQDs can be adjusted by their modification, allowing for the modulation of both the energy gap and TDM, which varies according to the geometric shape, functional group, and its position [45,46,47]. As such, the interaction between GQDs and glycine increases the dipolar moment and decreases the HOMO-LUMO energy gap, which optimizes its reactivity [48]. This is particularly evident in the ZTRI/glycine complex with sodium alginate, which exhibits high reactivity and stability [48]. In addition, the operation of GQDs with -NH_2_ groups promotes the adsorption of amino acids, which increases their applicability in the field of biomedicine [49]. In addition, research on super alkali-doped triphenylene and its derivatives functionalized with amino, hydroxyl, and thiol groups shows an increase in non-linear optical properties and optimized transparency in the ultraviolet region, providing the potential for progress in optics and electronics [50].

In the present study, GQDs with base structures of coronene were used, to which the groups -SH and -NH_2_ were subsequently added. The main objective was to perform a comparison of how the attachment of functional groups implies and improves the selectivity of GQDs. For this, we used calculations derived from the Density Functional Theory (DFT) with the method B3LYP/6-311+G(d,p), which provided us with a meticulous analysis of their properties in the selective detection of heavy metals such as Pb, Cr, and Cd in water bodies. This methodology aimed to deepen the understanding of the interrelationship between functional groups and specific pollutants to maximize the potential of GQD sensors in aquatic contexts.

## 2. Results and Discussion

### 2.1. Methods Selection

**Figure 2:**
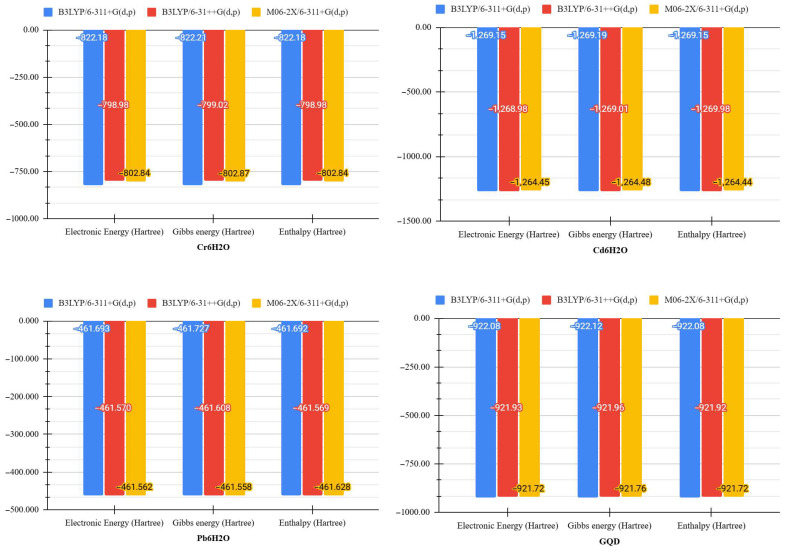

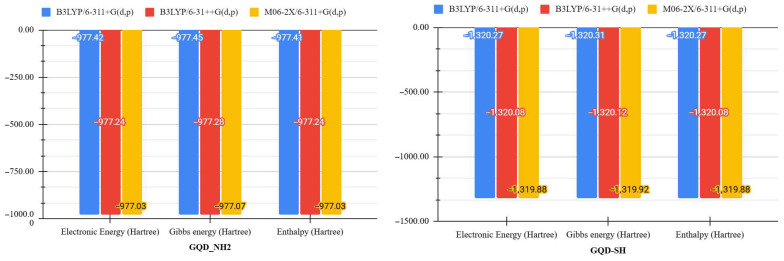
Comparative graphs of electronic energy (Hartree), Gibbs energy (Hartree), and enthalpy (Hartree), obtained by different computational methods (B3LYP/6-311+G(d,p), B3LYP/6-31++G(d,p), and M06-2X/6-311+G(d,p)) for the base systems.

Various configurations of methods and base sets such as B3LYP/6-311+G(d,p), B3LYP/6-311++G(d,p), and M06-2X/6-311+G(d,p) were implemented to evaluate the interaction of hydrated heavy metals with GQDs and GQD-M. The results, as presented in Table 1, show variations between these methods regarding electronic energy, Gibbs energy, entropy, and enthalpy. The B3LYP functional with the 6-311+G(d,p) basis set was selected due to its consistent generation of lower energy geometries compared to other configurations, indicating higher thermodynamic stability. This selection criterion was based on the evaluation of key physicochemical parameters, including Gibbs energy, electronic energy, enthalpy, and entropy. This behavior is also visually represented in Figure 2, where comparative bar charts highlight the differences in the thermodynamic parameters obtained with each computational functional and basis set. This phenomenon was particularly evident in the analyzed systems, where B3LYP/6-311+G(d,p) exhibited reduced energy and enthalpy values, indicating a positive and stable interaction between quantum dots and metal ions. The selection of B3LYP/6-311+G(d,p) was also more efficient from a computational perspective, facilitating a decrease in the costs related to calculations and maintaining a high degree of accuracy in characterizing the electronic and structural properties of carbon-based nanomaterial systems.

### 2.2. Representative Models

**Figure 3:**
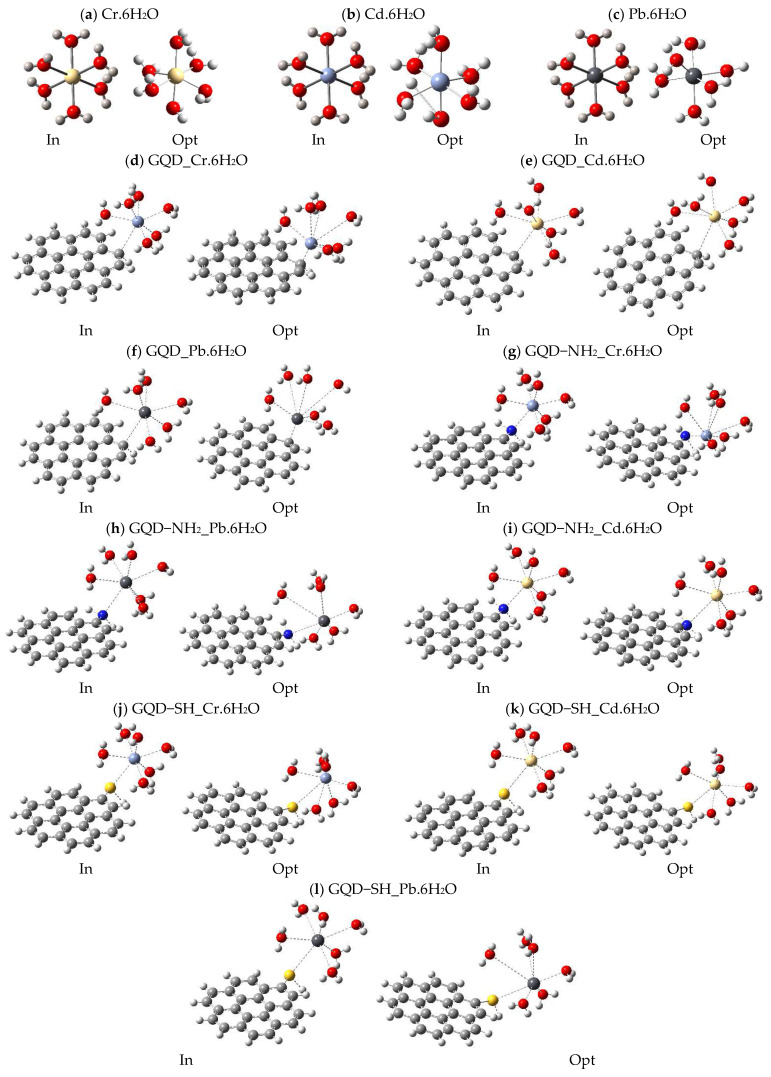
Initial (In) and optimized (Opt) structure with B3LYP/6-311+G(d,p). Black spheres represent carbon (C) atoms, white spheres represent hydrogen (H) atoms, red spheres nitrogen (N) atoms, yellow spheres are sulfur (S) atoms, gray spheres indicate lead (Pb), light blue spheres represent chromium (Cr), and beige spheres represent cadmium (Cd).

Essentially, the aim of this was to identify representative optimized models through the investigation of heavy metal interactions with GQD-M. Therefore, the simplified structure of coronene was chosen as a model structure due to its high stability and planar symmetry, characteristics that make it an ideal substitute for simulating GQDs in a computational context. Representative models were created through advanced configurations using the B3LYP/6-311+G(d,p) functional, which allowed us to obtain lower energy geometries and, therefore, greater thermodynamic stability. These configurations of the GQDs and the aforementioned hydrated metals are illustrated in Figure 3.

**Figure 4:**
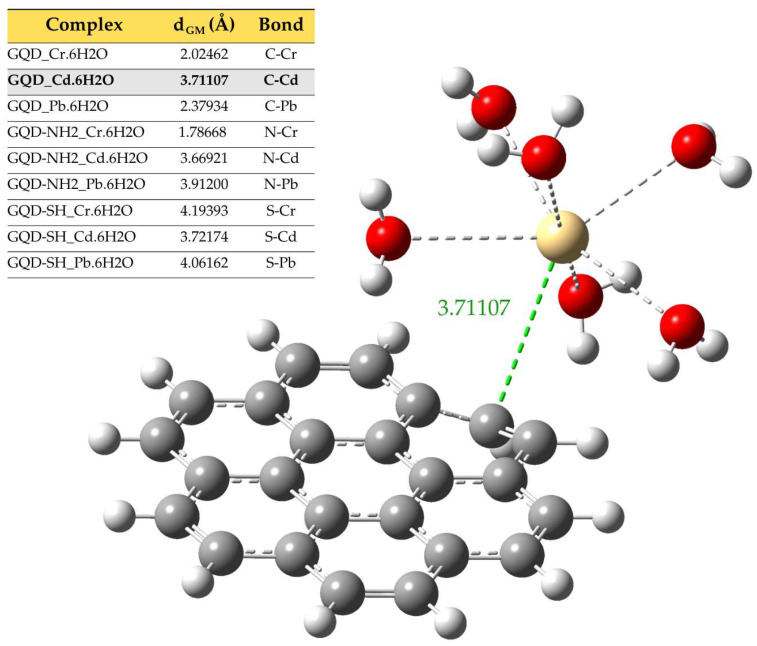
Structural representation of the adsorption distances (d_GM_) between the GQD and the metal complex.

In situations where a metal forms bonds with the functional groups found in GQDs, such an interaction can displace one or more water molecules embedded in the metal. To investigate this phenomenon more precisely, the B3LYP/6-311+G(d,p) functional was used in the context of DFT to model all the structures involved. This technique not only enabled the accurate simulation of the complex structures but also simplified the quantification of essential modifications in the electronic properties. The HOMO-LUMO band energy was evaluated, providing an indicator of the system’s stability and chemical reactivity. Furthermore, the molecular electrostatic potential was established, providing a comprehensive view of the charge distribution and the areas with the greatest electrostatic activity in the modeled complexes. On the other hand, Figure 4 shows the adsorption distances (dGM) derived from the optimized geometries, allowing a comparative visualization of the spatial focusing between the metal complexes and the GQD functional groups.

### 2.3. Analysis of Energy Gap (Eg) and Total Dipolar Moment (TDM) Parameters

Table 2 presents the relationship between GQDs and metal hexahydrates, and how their function influences the Eg and TDM. The Eg is a crucial indicator that affects the electronic stability and reactivity of a material. For the systems listed in Table 2, GQDs exhibit fairly stable Eg values, ranging from 0.07453 eV to 0.07692 eV, except for the species interacting with Pb.6H_2_O, indicating a more stable structure. As the -NH_2_ and -SH groups interact, a decrease in the Eg is noted. Specifically, the relationship between GQD-SH and Pb.6H_2_O shows values of 0.02175 eV. This decrease means that less energy is required to excite electrons from the highest occupied orbital (HOMO) to the lowest unoccupied orbital (LUMO). Consequently, electron transmission between the GQD and the metal is enhanced, increasing the system’s reactivity. This is crucial, since easier electron transfer is often linked to high catalytic activity and more efficient interactions with other chemical species in the environment.

The TDM indicates the polarization of the system, which demonstrates its ability to interact with polar molecules. In this scenario, a high TDM indicates that the GQD-SH not only attracts heavy metals such as Pb, generating more stable complexes, but could also affect the direction and dynamics of these interactions at the microscopic level, impacting the effectiveness of contaminant identification and adsorption. The interaction of the GQD with Cr.6H_2_O and Cd.6H_2_O shows moderately high TDM values, ranging from 3.28 D to 3.68 D, indicating effective polarization and significant potential for intermolecular interactions. On the other hand, the GQD-NH_2_ with Cd.6H_2_O presents considerably lower TDM values (0.53 D), which could affect its sensitivity in sensing applications. However, the GQD-SH with Cd.6H_2_O shows the highest TDM values, reaching 5.23 D, indicating a more marked polarization and, consequently, a greater ability to identify polar species.

### 2.4. Calculations of the Energy Gap Between Orbitals (HOMO-LUMO)

**Figure 5:**
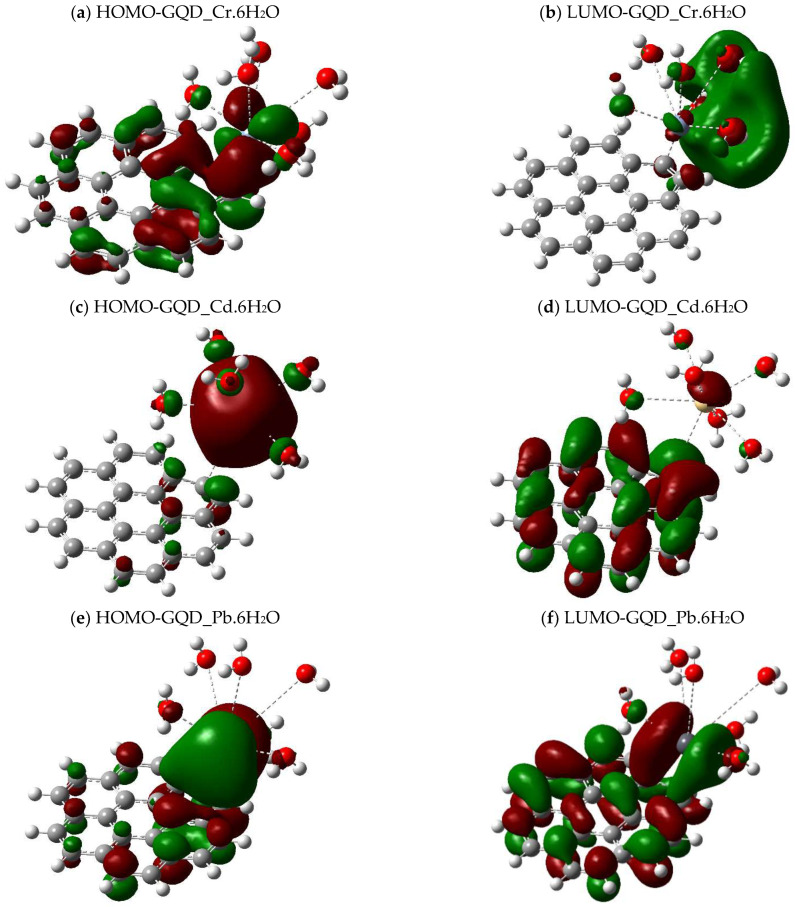

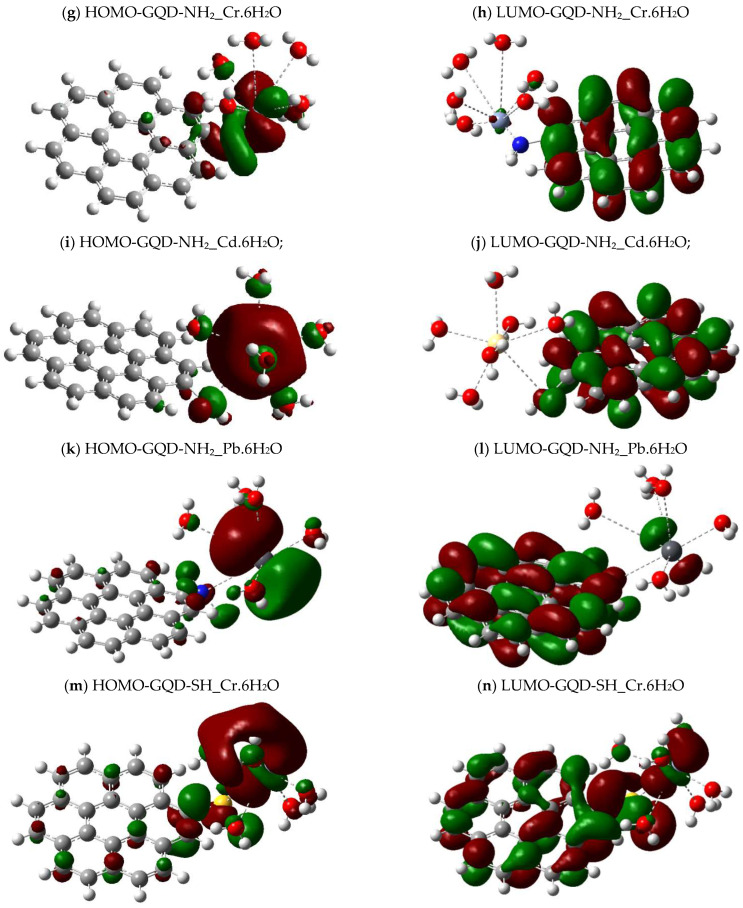

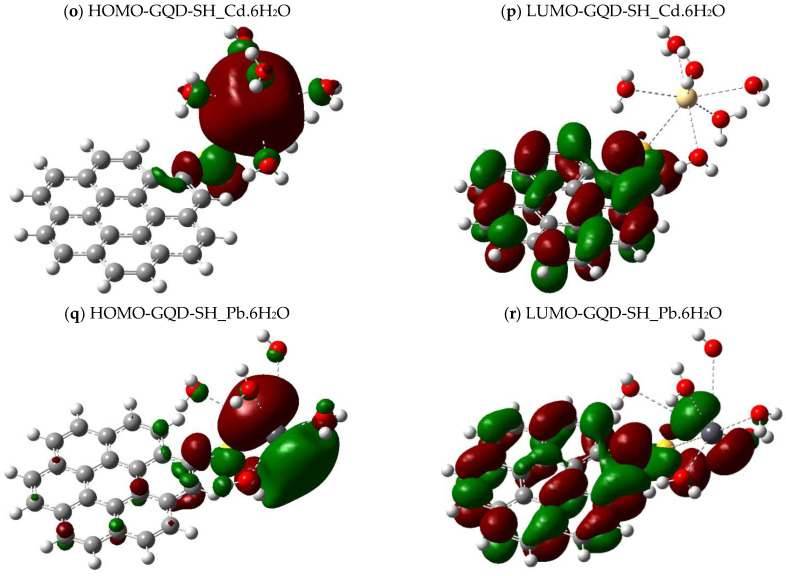
HOMO-LUMO band gap energy optimized with B3LYP/6-311+G(d,p).

The graphical representation of the molecular boundary orbitals (HOMO-LUMO) present in the GQD complexes that functionalize with groups -NH_2_ and -SH evidences the notable discrepancies in the electronic distribution and their interactions with metals such as Cr, Cd, and Pb. In the unfunctionalized GQD, the interaction with metals fluctuates according to species; in the Cr complex, HOMO exhibits a homogeneous distribution between the GQD and the metal, showing a stable charge transfer. On the other hand, LUMO is predominantly found in metal, demonstrating its function as an electron receptor (Figure 5a,b). In the Cd complex, HOMO concentrates on metal, which shows an intense electronic interaction. On the other hand, LUMO moves over the GQD, revealing its function as an electron acceptor (Figure 5c,d). In the case of Pb, both HOMO and LUMO exhibit a balanced distribution between GQDs and metal (Figure 5e,f).

The presence of the -NH_2_ group alters the electronic distribution in the GQD-NH_2_. With Cr, HOMO focuses on the metal and functional groups, ensuring a robust interaction, while LUMO moves extensively, simplifying the load transfer (Figure 5g,h). In the complex with Cd, HOMO is located on the metal, indicating its impact on stability; on the other hand, LUMO is redistributed over the GQD and functional groups, showing that Cd plays an electrophilic center role (Figure 5i,j). With Pb, HOMO manifests itself in metal and certain functional groups, while LUMO propagates through the GQD, increasing its sensitivity to environmental variations (Figure 5k,l). In GQD-SH, the interaction with metals is altered by sulfur doping. In the Cr complex, HOMO concentrates on the metal and functional groups, ensuring a robust electronic interaction, while LUMO propagates through the π system of the GQD, facilitating charge transfer (Figure 5m,n). With Cd, HOMO is present in the metal, indicating a restricted electron density, while LUMO is redistributed in the GQD and functional groups (Figure 5o,p). Finally, in the Pb complex, both HOMO and LUMO exhibit a balanced distribution, which favors charge transfer and gives this material considerable potential for electronic and sensory applications (Figure 5q,r).

### 2.5. Electrostatic Potential Map (MEP)

**Figure 6:**
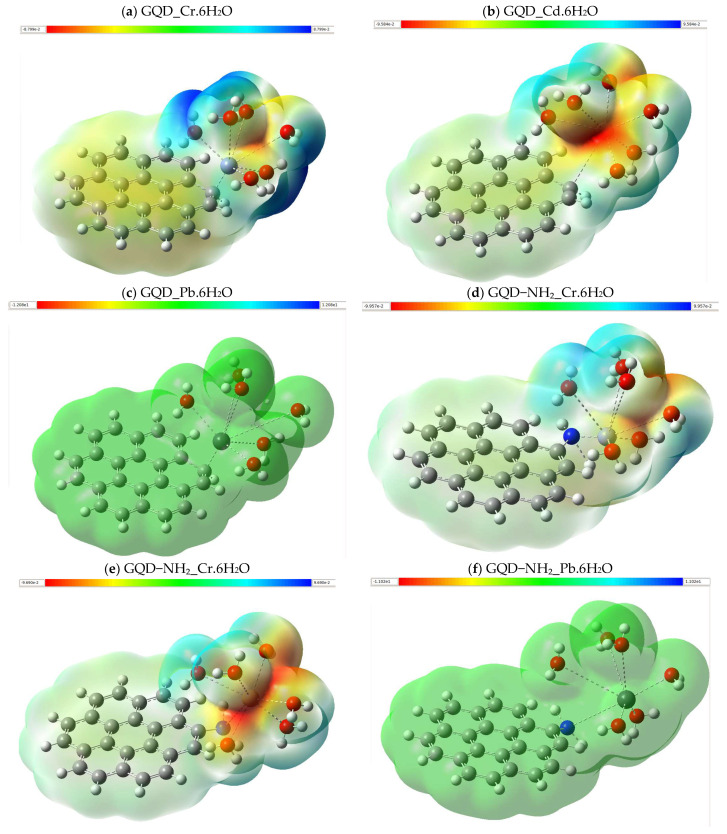

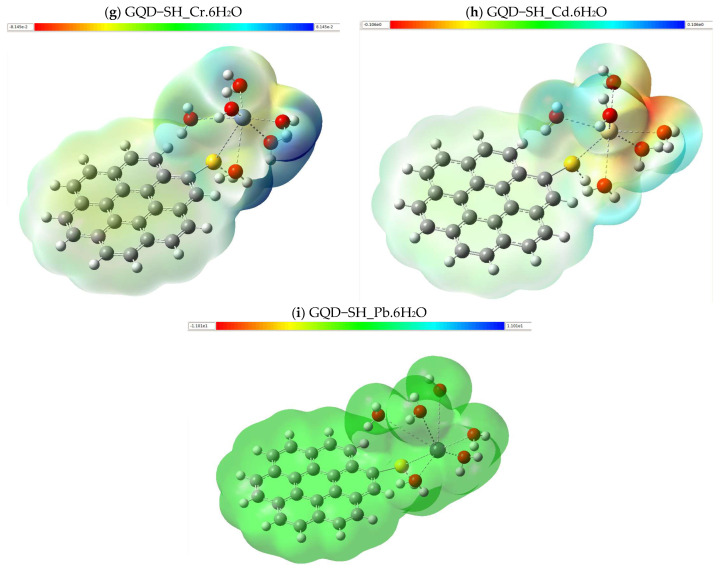
Electrostatic potential maps of the structures optimized with B3LYP/6-311+G(d,p).

The MEPs of the different GQD-based systems are shown below, these diagrams provide a visual representation of the electrostatic charge distribution and reactivity of each system. The various changes in the electrostatic potential maps of GQDs modified after the adsorption of Cr, Cd, and Pb indicate significant differences in charge distribution; these are important for understanding electronic interactions and their applicability in sensors. The adsorption of metals induces a redistribution of electron density on the surface of the GQD, altering the regions of higher and lower electrostatic potential. Therefore, variations in the MEP can modify the optoelectronic behavior of the GQD, adjusting its energy gap and its response to external stimuli. In the non-functionalized species, GQD_Pb.6H_2_O presents a relatively uniform electrostatic potential distribution, mainly highlighting the green tone. This tone indicates an average electrostatic charge intensity, indicating that the electron density is distributed evenly over the entire surface of the system (Figure 6c). The lack of clearly positive or negative areas indicates that lead does not cause a noticeable reallocation of the electronic load compared to other metals. On the other hand, GQD_Cr.6H_2_O shows a more marked load differentiation, manifested in the existence of areas of intense blue around the Cr and red areas in the oxygenates. Blue points to areas with a high negative electron density, while red points to an accumulation of positive charge, suggesting a high polarization. The electronically strong interaction and a high degree of polarization within the complex are indicated by the significant attraction of Cr towards the electron density metal. Similarly, GQD_Cd.6H_2_O shows a charge distribution similar to that of Pb, although it presents a greater presence of positive charge areas near the Cd. The existence of these areas indicates that Cd redistributes the load more equitably than Cr, but more noticeably than Pb. Increased polarization around Cd could simplify specific interactions with negatively charged molecules, which could be used for the creation of selective sensors for cadmium identification.

In -NH_2_-powered species, GQD-NH_2__Cr.6H_2_O retains a similar loading pattern to the non-powered species, although with a decrease in blue zone intensity. This indicates that activity with -NH_2_ increases the negative charge in Cr areas, possibly due to the ability of the amino group to provide electrons (Figure 6d). Increasing the negative charge in Cr could enhance the system’s ability to identify metal species, since any interaction with metallic cations could cause noticeable changes in the load distribution. The charge redistribution in GQD-NH_2__Cd.6H_2_O is similar to that of GQD-NH_2__Cr.6H_2_O (Figure 6e), indicating that the -NH_2_ operation influences the interaction with both metals similarly. On the other hand, GQD-NH_2__Pb.6H_2_O maintains a fairly uniform potential distribution (Figure 6f), indicating that Pb is not significantly affected by -NH_2_, which could restrict its ability to be detected through changes in load distribution. In -SH species, GQD-SH_Cr.6H_2_O shows the highest load differentiation of all studied species. There is an increase in the areas of intense blue in the Cr region and red in the oxygens present in aquatic molecules, in contrast to Figure 6d. This shows that the -SH group improves charge separation compared to -NH_2_, possibly due to its greater ability to interact with metal by covalent bonds and induction effects. This marked load differentiation could improve the sensitivity of -SH-functionalized systems to detect Cr through modifications in the electrostatic potential distribution. In addition, GQD-SH_Cd.6H_2_O shows a more even charge distribution, although there are still some areas of polarization in the Cd region (Figure 6h). This indicates that operation with -SH does not have as significant an effect on charge redistribution as it does with Cr, which could imply that the identification of Cd in these systems requires additional methods such as spectroscopic techniques. Finally, GQD-SH_Pb.6H_2_O shows a more uniform load distribution among all species, highlighting the green color without evidence of high-polarization zones. This indicates that Pb has a limited ability to cause considerable charge separations, which could complicate its direct electrostatic identification. However, their identification could be addressed through specific interactions with functional groups or changes in the fluorescence of the system.

## 3. Materials and Methods

### 3.1. Computational Details

In this investigation, theoretical calculations were used, using the Density Functional Theory (DFT) [51,52] to analyze the insertions of hydrated heavy metals (Cr, Cd, and Pb) with graphene quantum dots (GQDs) functionalized with thiol (-SH) and amino (-NH_2_) groups. Given the complexity and size of graphene quantum dots, coronene, a well-defined polyaromatic hydrocarbon known for its planar symmetry and high stability, was chosen as the simplified model structure for these GQDs [53]. This was justified due to the similarity in the π-conjugated systems between the coronene and the GQDs, which allowed a representative approximation in this computational analysis. This configuration also incorporated a conjugate ring arrangement that could increase the GQD’s sensitivity to heavy metals by facilitating more efficient charge transfer [54,55]. DFT calculations were performed using Gaussian software 16 A.03 [56]. The following method/base configurations were implemented: B3LYP/6-311+G(d,p), B3LYP/6-311++G(d,p), and M06-2X for base structures. B3LYP/6-311+G(d,p) was chosen because it produces lower energy geometries, suggesting greater thermodynamic stability and lower computational costs. In addition, this level of theory has evidenced a more precise description of the electronic structure in carbon-based nanosystems, achieving a more accurate representation of their electronic and geometric characteristics [57]. Although the primary objective of this study was to investigate the interaction between modified GQDs and the heavy metals in question, common inorganic ions present in real wastewater, such as Na^+^, K^+^, and Ca^2+^, were not included in this computational model. This focus allowed us to distinguish the electrical influence of heavy metal ions without other foundational cations interfering. Although these ions were relevant, they were excluded to maintain the feasibility of quantum mechanical calculations and ensure an in-depth analysis of the selected systems. On the other hand, since in aqueous systems, heavy metals are predominantly in hydrated form, six water molecules were explicitly incorporated into the modeling of metal complexes to assess their influence on the adsorption process [58,59,60].

### 3.2. Evaluation of Electronic Properties

Analysis of the electronic properties of GQDs functionalized with -SH and -NH_2_ groups interacting with hydrated heavy metals provides a better understanding of their sensor behavior. Therefore, the most relevant properties discussed in this study are presented.

#### 3.2.1. Calculating the Energy Gap (*Eg*)

The energy gap (*Eg*) between the HOMO-LUMO frontier orbitals provides relevant information about the reactivity of the system. A lower *Eg* implies a greater ease of electronic excitation and, likewise, a greater interaction capacity with chemical species such as metals. *Eg* is calculated from the energy difference Equation (1).

(1) $$Eg=E_{LUMO}-E_{HOMO}$$

#### 3.2.2. Total Dipole Moment (TMD)

The total dipole moment is a vector parameter that determines the charge distribution within the molecule. Higher TDM values imply greater polarity, thus favoring interaction with polar molecules or metal in solution.

#### 3.2.3. Molecular Electrostatic Potential (MEP)

Electrostatic potential maps show the charge distribution on the molecule’s surface, revealing nucleophilic and electrophilic regions. This is useful for visualizing preferential interaction sites with metals. MEPs were generated for each optimized complex using Gaussian 16 A.03 software. Red areas indicated regions with a higher proportion of electrons (negative potential), while blue areas represented regions with a lower proportion of electrons (positive potential).

This analysis did not include adsorption energy calculations, as the primary goal was to assess the suitability of electronic descriptors to predict reactivity and the ability to selectively identify functionalized GQDs. Instead of determining adsorption energies, we examined the HOMO-LUMO gap, total dipole moment, and electrostatic potential maps, which provided additional information about interaction potential, charge polarization, and molecular reactivity.

## 4. Conclusions

The functionalization of graphene quantum dots (GQDs) with thiol (-SH) and amino (-NH_2_) groups focused on enhancing the selective identification of heavy metals in wastewater. Using the B3LYP/6-311+G(d,p) Density Functional Theory (DFT) method, the interaction between the functionalized GQDs and hydrated metal ions such as Cr, Cd, and Pb was simulated. The findings showed that the inclusion of these functional groups not only improved the ability to detect heavy metals but also resulted in a reduction in the Eg and an increase in the TDM. These modifications indicated the increased reactivity and interaction capacity of GQDs with metals, particularly highlighting the polarization and identification of Pb in GQDs functioning with -SH groups. The functional -SH and -NH_2_ groups not only increased the sensitivity and selectivity toward heavy metals but also enhanced the electronic and optical characteristics of GQDs, making them ideal for use in environmental sensors. These findings highlight the need for further investigation into the functioning of GQDs and other nanomaterials to develop more efficient and accurate detection technologies in the fight against heavy metal pollution in wastewater.

Despite the results achieved, it is crucial to consider several limitations in this study. First, the theoretical level employed, specifically the B3LYP/6-311+G(d,p) method, although widely accepted for investigations of carbon-based systems, may not have fully encompassed all electronic and structural effects in more complex systems. Furthermore, the lack of solvent impact in the simulations may have affected the accuracy of the results, since interactions in a real aqueous environment can vary considerably from the ideal conditions assumed in the computational model. Furthermore, the GQD surface model employed may not have accurately represented the geometry and reactivity of GQDs under experimental conditions, which could have restricted the usefulness of the findings. To address this study’s limitations, we suggest that future studies experimentally verify the results obtained through computational models, which will facilitate the verification of the effectiveness of functionalized GQDs in identifying heavy metals in real-world situations. Additionally, we recommend investigating other functional groups to increase the selectivity and sensitivity of GQDs in contaminant identification. Finally, a more in-depth study of the detection mechanism, including kinetic investigations and molecular-level interactions, could provide a more detailed understanding of the interaction of GQDs with heavy metals and improve their performance in practical applications

## 5. Supplementary Information

Global descriptors, which are parameters that encompass various properties of molecules, provide fundamental information about their ability to interact effectively with their chemical environment. This information is essential for predicting and understanding their behavior in a wide variety of contexts, such as chemical reactions, intermolecular interactions, and electron transfer processes. The descriptors considered for analysis and evaluation include the following elements and characteristics:

- Ionization energy (I): Defines the energy required to extract an electron from the HOMO of the system in its ground state. A high *I* value indicates a high electronic stability against ionization, while low values indicate greater reactivity and ease in donating electrons [61,62].
- Electron affinity (A): Refers to the energy released when the system accepts an extra electron in the LUMO. A high A indicates a good performance as an electron acceptor, which is significant in interactions with heavy metals that can function as partial donors in coordination complexes [62,63,64,65,66].
- Chemical hardness (η): It evaluates the system’s ability to withstand changes in its electron density. High-hardness systems show lower chemical reactivity, indicating greater stability. In contrast, low hardness indicates a greater ability of the system to interact with external elements such as hydrated metal ions [61,63].
- Electronegativity (χ): This global parameter, calculated as the average between I and A, characterizes the system’s ability to attract electrons to itself during a chemical interaction. High electronegativity leads to a stronger polar interaction, which favors adsorption or complexation processes with species [64,65].
- Chemical potential (µ): It represents the system’s inherent tendency to acquire or donate electrons based on its electronic configuration and its interaction with other chemical elements in its environment. Significantly low *µ* values indicate that the molecule has a greater natural tendency to donate electrons, while notably high values suggest a more pronounced acceptor nature. This molecular descriptor makes it possible to predict in advance the preferential interaction of functionalized derivative-quality graphene with electrophilic metal ions [63,66].

## Figures and Tables

**Table 1:** Comparison of methods/bases in the thermodynamic evaluation of molecules interacting with hydrated metal.

Molecule	DFT Functional/Basis Set	Electronic Energy (Hartree)	Gibbs Energy (Hartree)	Enthalpy (Hartree)	Entropy (Cal/mol-Kelvin)
Cr.6H_2_O	B3LYP/6-311+G(d,p)	−82 2.178	−822.210	−822.177	69.886
	B3LYP/6-31++G(d,p)	−798.982	−799.015	−798.981	69.899
	M06-2X/6-311+G(d,p)	−802.841	−802.874	−802.840	69.898
Cd.6H_2_O	B3LYP/6-311+G(d,p)	−1269.154	−1269.187	−1269.153	70.871
	B3LYP/6-31++G(d,p)	−1268.983	−1269.014	−1269.980	70.812
	M06-2X/6-311+G(d,p)	−1264.445	−1264.478	−1264.444	70.922
Pb.6H_2_O	B3LYP/6-311+G(d,p)	−461.692	−461.727	−461.692	74.608
	B3LYP/6-31++G(d,p)	−461.570	−461.608	−461.569	81.336
	M06-2X/6-311+G(d,p)	−461.562	−461.558	−461.628	80.405
GQD	B3LYP/6-311+G(d,p)	−922.084	−922.120	−922.0820	77.340
	B3LYP/6-31++G(d,p)	−921.923	−921.961	−921.924	77.339
	M06-2X/6-311+G(d,p)	−921.721	−921.757	−921.720	77.334
GQD-NH_2_	B3LYP/6-311+G(d,p)	−977.415	−977.453	−977.414	83.357
	B3LYP/6-31++G(d,p)	−977.238	−977.277	−977.237	83.335
	M06-2X/6-311+G(d,p)	−977.034	−977.073	−977.033	83.260
GQD-SH	B3LYP/6-311+G(d,p)	−1320.270	−1320.311	−1320.269	88.119
	B3LYP/6-31++G(d,p)	−1320.080	−1320.121	−1320.079	88.101
	M06-2X/6-311+G(d,p)	−1319.881	−1319.922	−1319.880	87.875

**Table 2:** The energy gap (Eg) and the total dipolar moment (TDM).

Structure	Interaction	Eg (eV)	TDM (D)
GQD	Cr.6H_2_O	0.07453	3.286890
	Cd.6H_2_O	0.04286	3.286890
	Pb.6H_2_O	0.07692	5.689753
GQD-NH_2_	Cr.6H_2_O	0.06823	4.252212
	Cd.6H_2_O	0.03571	0.533976
	Pb.6H_2_O	0.02140	2.008093
GQD-SH	Cr.6H_2_O	0.01781	5.231810
	Cd.6H_2_O	0.03016	3.514984
	Pb.6H_2_O	0.02175	4.066719

## Data Availability

The data are partially available due to confidentiality at the chemical plant where the evaluations were carried out.
